# Efficient Adsorption-Photocatalytic Removal of Tetracycline Hydrochloride over Octahedral MnS

**DOI:** 10.3390/ijms23169343

**Published:** 2022-08-19

**Authors:** Jing Guo, Tingting Liu, Hao Peng, Xiaogang Zheng

**Affiliations:** 1 College of Chemistry and Chemical Engineering, Yangtze Normal University, Chongqing 408100, China; 2 College of Chemistry and Chemical Engineering, Neijiang Normal University, Neijiang 641100, China

**Keywords:** MnS, octahedral structure, tetracycline hydrochloride, adsorption-photocatalysis

## Abstract

To disclose the effect of crystal plane on the adsorption-photocatalytic activity of MnS, octahedral MnS was prepared via the hydrothermal route to enhance the adsorption and photocatalytic efficiencies of tetracycline hydrochloride (TCH) in visible light region. The optimal MnS treated at 433 K for 16 h could remove 94.83% TCH solution of 260 mg L^−1^ within 180 min, and its adsorption-photocatalytic efficiency declined to 89.68% after five cycles. Its excellent adsorption-photocatalytic activity and durability were ascribed to the sufficient vacant sites of octahedral structure for TCH adsorption and the feasible band-gap structure for visible-light response. In addition, the band gap structure (1.37 eV) of MnS with a conduction band value of −0.58 eV and a valence band value of 0.79 eV was favorable for the generation of O^2−^, while unsuitable for the formation of OH. Hence, octahedral MnS was a potential material for the removal of antibiotics from wastewater.

## 1. Introduction

Tetracycline hydrochloride (TCH) is one of the typical tetracycline antibiotics for the treatment and prevention of animal and human diseases [1,2]. Unfortunately, the overuse and poor digestion of TCH induce to the serious discharge into soil and aqueous environments with the concentration range varied from ng L^−1^ to μg L^−1^, forming the antibiotic resistance genes of bacterial flora [3,4,5]. This consequently results in life-threatening infections [6]. Hence, various approaches such as physical removal, chemical degradation, and biological treatment are intensively explored to remove and degrade TCH residues from wastewater before being released into the ecological system. Although the physical routes such as sedimentation, adsorption, filtration, and flocculation can separate TCH residues from an aqueous environment, they generate the contaminated adsorbents [7,8]. In addition, the biological methods seriously bring the active organisms into aquatic system, disrupting the ecological balance of biomes and even ecosystem damage [9]. The chemical approaches such as chlorination, ozonation, and Fenton’s oxidation suffer from the unintended damages of non-target organisms, low selectivity, and high operating costs [10,11,12]. Hence, the low-cost and high selective strategies are still exported to achieve the complete mineralization of antibiotic residues. In recent years, photocatalysis has garnered wide attention in environmental remediation and energy extraction [13,14,15].

Photocatalysis is an easilyattainable and strong redox reaction system with the dissolved oxygen as powerful oxidant and the solar light as energy source, converting the organic pollutants into innocuous molecules such as H_2_O and CO_2_ [16,17]. In recent years, various photocatalysts with proper band-gap structure, appropriate spectral response range, and sufficient active sites have been explored for photocatalytic degradation of pollutants [18,19,20]. Typically, metal sulfides such as MoS_2_ [21], CdS [22], ZnS [23], CuS [24], In_2_S_3_ [25], NiS [26], FeS [27], Ag_2_S [28], and Ga_2_S_3_ [29] are intensively investigated in photocatalytic reaction. Heterojunctions formed between different metal sulfides are further confirmed to overcome the quick annihilation of charge carriers and inferior optical utilization of single component [30,31]. In addition, Mn-based oxide and sulfide have attracted enormous attention as the efficient photocatalysts for environmental treatment and photocatalytic synthesis [32]. As a p-type semiconductor, MnS with a wide bandgap (3.7 eV) is widely applied in solar cells serving as optical storage and mass memories, solar selective coatings, and short-wavelength optoelectronic materials. Traditionally, MnS integrated with other metal sulfides such as CdS [33,34,35], (In_x_Cu_1−x_)_2_S_3_ [36], In_2_S_3_ [37], Ag_2_S [38], and Ni_3_S_2_ [39] are confirmed as the excellent visible-light heterojunctions for enhancing the photocatalytic activity in visible light region. MnS combined with metal oxides such as TiO_2_ [40] and WO_3_ [41] are also fabricated to strengthen the visible-light absorption capacity and to accelerate the transfer and separation of charge carriers. For instance, MnS-CdS solid solution exhibits the proper band-gap structure and high crystallinity, enhancing the H_2_ evolution capacity and photocatalytic stability [42,43,44]. However, there are few works discussing the effect of crystal structure on the adsorption-photocatalytic activity of pure MnS.

The polymorph structures such as stable rock salt (α-MnS), metastable zinc blende (β-MnS), and wurtzite (γ-MnS) greatly affect the photocatalytic performance of MnS [45]. For example, α-MnS/Bi_2_MoO_6_ exhibits the excellent visible-light photocatalytic activity and stability of CO_2_ reduction [46]. The mixed polymorphs of α-MnS and γ-MnS are favorable for the high photoelectrochemical and photocatalytic activities of MnS coated with MoS_2_ nanolayers [47]. Due to the naked (200) plane, cubic α-MnS treated in hydrazine hydrate-assisted hydrothermal system presents the H_2_ evolution rate of 223.4 mmol·h^−1^ in visible light region [48]. Hence, α-MnS has been devoted great efforts to prepare the novel architectures with promising photocatalytic activity. Considering the feasible conversion of β-MnS and γ-MnS into α-MnS at high temperature, the hydrothermal and solvothermal approaches are employed to synthesize the various MnS nanocrystals such as spheres [49], boxes [50], flowers [51], rods [52], dandelion [53], cubes [54], corals [55], stars [56], biopods [57], and multipods [58]. However, the fabrication of hierarchical α-MnS, such as octahedral structure via a simple approach, still suffers from a serious challenge.

This work focused on the synthesis of octahedral α-MnS via a hydrothermal route to investigate the adsorption-photocatalytic performance in TCH removal. The constructed octahedral structure and tuned band-gap state of MnS favored the visible-light response, leading to the excellent adsorption-photocatalytic efficiency. The removal efficiency of TCH over MnS was affected by pH, hydrothermal temperature, hydrothermal time, and inorganic salts.

## 2. Results and Discussion

**Figure 1:**
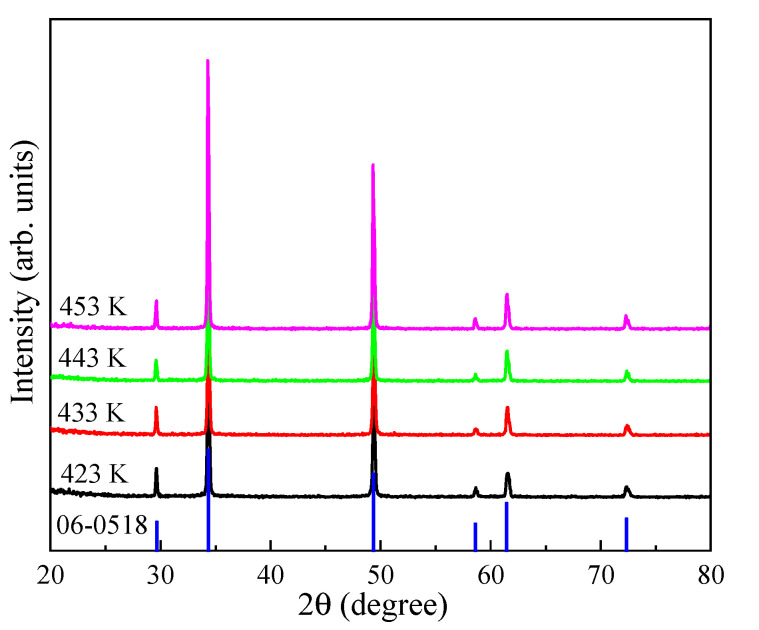
XRD patterns of MnS treated at different temperatures.

**Figure 2:**
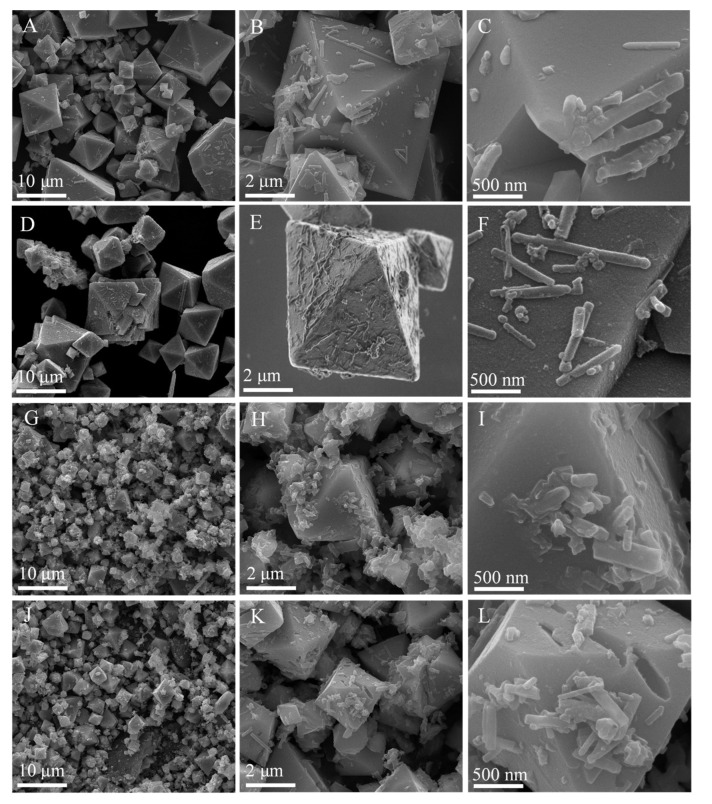
SEM images ofMnS treated at 423 K (**A**–**C**),433 K (**D**–**F**), 443 K (**G**–**I**), and 453 K (**J**–**L**).

XRD patterns of MnS treated at various temperature and time were performed in Figure 1 and Figure S1. These samples exhibited the typical rock salt α-MnS phases (JCPDS, 06-0518), of which the (111), (200), (220), and (222) facetswere located at 29.52°, 34.32°, 49.19°, and 61.48°, respectively [34,46]. The crystallinity of these MnS phases increased with the increasing hydrothermal temperature and time. In the solvothermal system, thiourea was decomposed into S^2−^ ions, then quickly reacted with Mn^2+^ ions to form MnS crystal nucleus, and further assembled into the desired structure with the assistance of surfactant [48,49]. Notably, the hydrothermal temperature and time greatly affected the morphologies of α-MnS (Figure 2 and Figure S2). α-MnS treated at 423 K was the octahedral structure with the irregular particle size, and there were many MnS nanoparticles and nanorods loading on the surface of α-MnS (Figure 2A–C). For comparison, the morphology of octahedral α-MnS treated at 433 K became much more regular, while part of α-MnS particles aggregated into clusters (Figure 2D–F) [48,54]. The side length of a typically octahedral structure was around 4 μm (Figure 2E). When increased to 443 K, a large amount of large octahedral particles were dissolved into the smaller and irregular particles of around 2 μm (Figure 2G–I). What is worse, there were many more nanoparticles existing on the surface of α-MnS treated at 453 K (Figure 2J–L). When the hydrothermal time climbed from 14 to 20 h, the octahedral MnS was destroyed into nanoparticles, and then aggregated into clusters (Figure S2).

**Figure 3:**
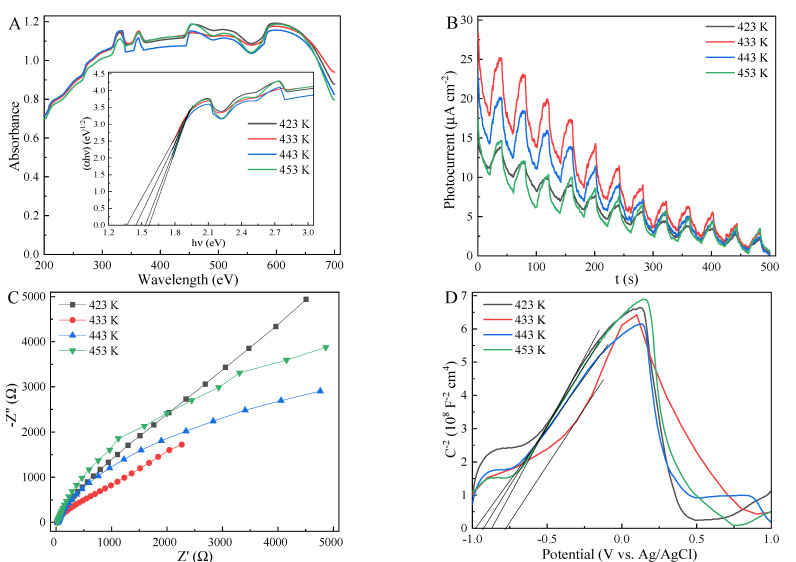
UV-vis DRS (**A**), photocurrent response curves (**B**), electrochemical impedance spectra (**C**), and Mott-Schottky curves (**D**) of MnS treated at different temperatures.

UV-vis DRS results (Figure 3A and Figure S3) indicated that MnS exhibited the strong visible-light response, which was slightly affected by the hydrothermal temperature and time. Based on the linearized Kubelka–Munk equation, the estimated band-gap energy (Eg) values of MnS were also mildly influenced by the hydrothermal temperature and time (Table S1) [55,59]. Notably, the Eg value of α-MnS treated at 433 K for 16 h was 1.37 eV. Among these MnS samples treated at various temperatures, the MnS obtained at 433 K presented the strongest photocurrent (Figure 3B). However, the photocurrent intensities of MnS samples decreased with the increasing radiation time. It is indicated that the photo-induced holes and electrons of MnS are likely to recombine in the irradiation process [47,60]. Due to the unique octahedral structure of MnS, the photoinduced electron-hole pairs are efficiently at the edge sites and the coarse plane of MnS, the combination of photo-generated electron-hole pairs could be efficiently restrained [33,34]. The smallest radius of electrochemical impedance spectra (Figure 3C) was obtained by MnS treated at 433 K for 16 h, meaning the highest efficiency of charge carveries transfer and the lowest resistance of surface layer [22,25]. Considering the flat-band potentials (EFB) close to the Fermi level (Ef) (Figure 3D and Figure S4), the EFB values of MnS samples estimated by the Mott-Schottky equation ranged from −0.778 to −0.974 V (Table S1). Hence, the calculated ECB and EVB of MnS treated at 433 K for 16 h were −0.58 and 0.79 eV, respectively.

**Figure 4:**
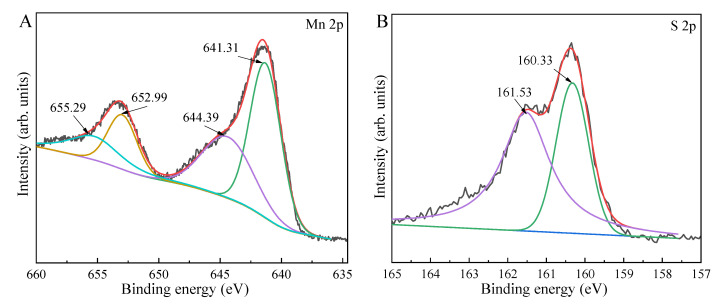
Mn 2p (**A**) and S 2p (**B**) XPS spectra of MnS treated at 433 K.

**Figure 5:**
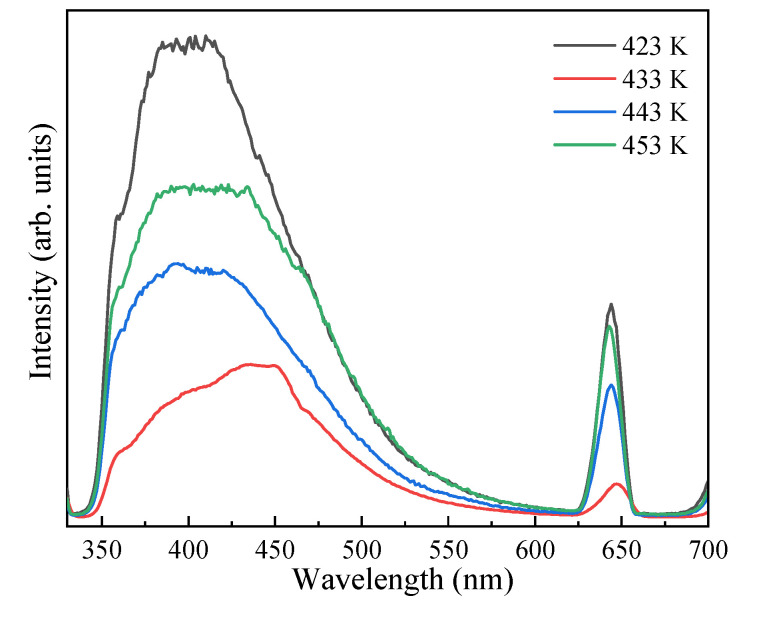
PL spectra of MnS treated at different temperatures.

For MnS treated at 433 K for 16 h, the split peaks of Mn 2p at 652.99 and 641.31 eV were respectively ascribed to Mn 2p1/2 and Mn 2p3/2 of Mn^2+^, and their corresponding satellite peaks located at 655.29 and 644.39 eV (Figure 4A) [33,59]. The dissected peaks of S 2p at 161.53 and 160.33 eV (Figure 4B) were ascribed to S 2p1/2 and S 2p3/2, respectively. However, the tested atomic ratio of Mn/S was lower than 1:1, indicating the formation of S defects in octahedral MnS. The Zeta potential plots (Figure S5) indicated that the pH range of 3.00~9.36 and 10.75~11.00 induced to the negative surface discharge of MnS treated at 433 K for 16 h, while the pH range of 9.36~10.75 resulted in the positive surface discharge. Previous works [60,61] reported that TCH had three species, including cationic (TCH^3+^) at pH < 3.3, zwitterionic at pH of 3.3~7.7, and anionic (TCH^−^ and TC^2−^) at pH > 7.7, respectively. Hence, the obtained MnS could efficiently adsorb TCH at pH of 3.3~10.75 via the electrostatic attraction, maximizing the adsorption efficiency of TCH [27,38]. PL spectra (Figure 5) suggested that the PL intensity of MnS decreased and then increased with an increase in hydrothermal temperature. For comparison, MnS treated at 433 K exhibited the lowest PL intensity, meaning the efficient transfer and separation of charge carriers.

**Figure 6:**
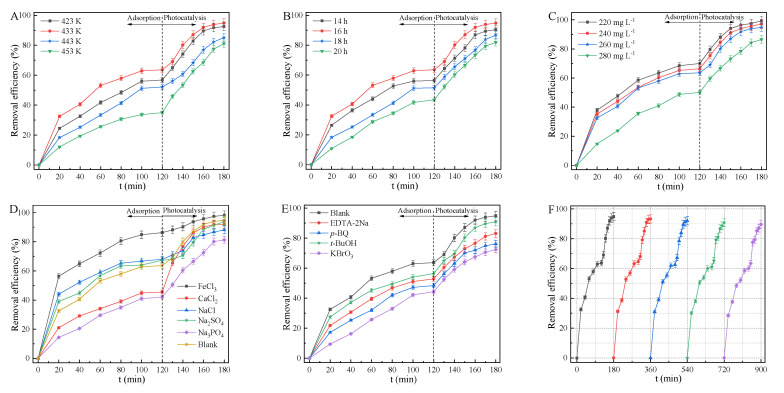
Effects of hydrothermal temperature (**A**), time (**B**), TCH concentration (**C**), inorganic salts (**D**), and quenchers (**E**) on the adsorption-photocatalytic activity of MnS, and durability of MnS (**F**) for TCH removal.

The adsorption and photocatalytic behaviors of MnS were carried out for the removal of TCH in visible light region (Figure 6). The adsorption-photocatalytic activity of MnS was greatly affected by pH, hydrothermal temperature, hydrothermal time, and inorganic salts [47,55,62]. Due to the electrostatic attraction between obtained MnS and TCH for efficient adsorption of TCH from aqueous solution at pH of 3.3~10.75, the removal efficiency of TCH was greatly affected by photo-induced radicals such as O^2−^ and OH. The high pH is not favorable for the formation of OH in photocatalytic system, leading to the inferior photocatalytic activity of photocatalysts [13,63]. Hence, the optimal pH was 4.36 for TCH removal over MnS. With the increasing hydrothermal temperature, the adsorption and photocatalysis efficiencies of MnS for TCH solution of 260 mg L^−1^ (Figure 6A) increased and then decreased within 180 min, of which the best removal efficiency of TCH over MnS treated at 433 K for 12 h was 94.83%. In contrast with the reported works, the removal efficiency of MnS (Table S2) was better, suggesting that octahedral MnS was a potential adsorption-photocatalytic material for wastewater treatment [63,64]. The adsorption-photocatalytic efficiency of MnS (Figure 6B) increased and then decreased with an increase in hydrothermal time. The main reason was that the hydrothermal temperature and time intensively affected the crystallinity of MnS. The high hydrothermal temperature and time induced to the decreased numbers of S defect sites, suppressing the adsorption and photocatalysis activity of MnS [49,51]. TCH molecules diffused from aqueous solution to vacant sites of bulk surface, meanwhile the decomposed intermediates and products escaped from active sites of solid surface [36,47,61]. This process was seriously affected by the high concentration of TCH. As plotted in Figure 6C, the adsorption-photocatalytic efficiency of MnS declined with the climbing TCH concentration ranging from 220 to 280 mg L^−1^.

**Figure 7:**
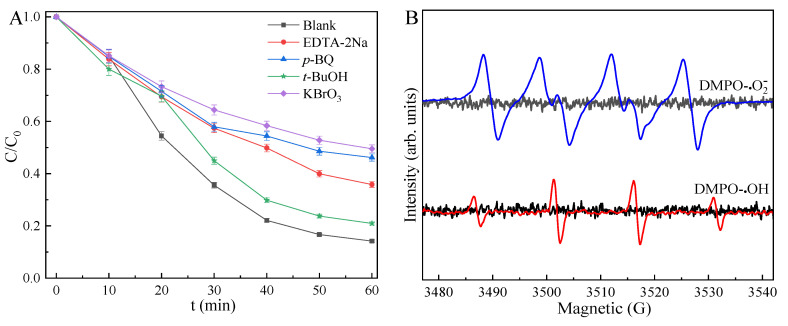
Photocatalytic quenching (**A**) and ESR spectra (**B**) of MnS.

The adsorption-photocatalytic activity of MnS was seriously affected by inorganic salts, especially in industrial application of wastewater treatment [23,24]. In contrast with MnS alone for TCH removal, inorganic salts such as CaCl_2_, Na_2_SO_4_, and Na_3_PO_4_restrained, while FeCl_3_ promoted the removal efficiency of TCH (Figure 6D). These ions competitively adsorbed on the vacant sites of MnS, reducing the number of active sites for TCH adsorption and photocatalysis, especially Na_3_PO_4_. However, FeCl_3_ could enhance the adsorption activity of MnS, Fe^2+^/Fe^3+^ redox couples also favored the photocatalytic reaction, inducing to the excellent photocatalytic activity [14,64]. Quenchers such as EDTA-2Na. p-BQ, t-BuOH, and KBrO_3_ were not favorable for the adsorption and photocatalysis of MnS (Figure 6F). It is indicated that the photo-induced h+, O^2−^, OH, and e-species played the main roles in visible-light degradation of TCH, especially the vital role of e^−^ for MnS-assisted photocatalytic reaction (Figure 7A) [44,60]. ESR results (Figure 7B) also suggest that O^2−^ and OH were responsible for the excellent photocatalytic activity of MnS [62,63]. However, the long-term photo-corrosion resulted in the decreased amount of these radical species during the photocatalytic process, leading to the inferior photocatalytic efficiency of MnS. As presented in Figure 6F, the removal efficiency of TCH over MnS decreased from 94.83% to 89.68% after five cycles, exhibiting the excellent durability of octahedral MnS.

**Figure 8:**
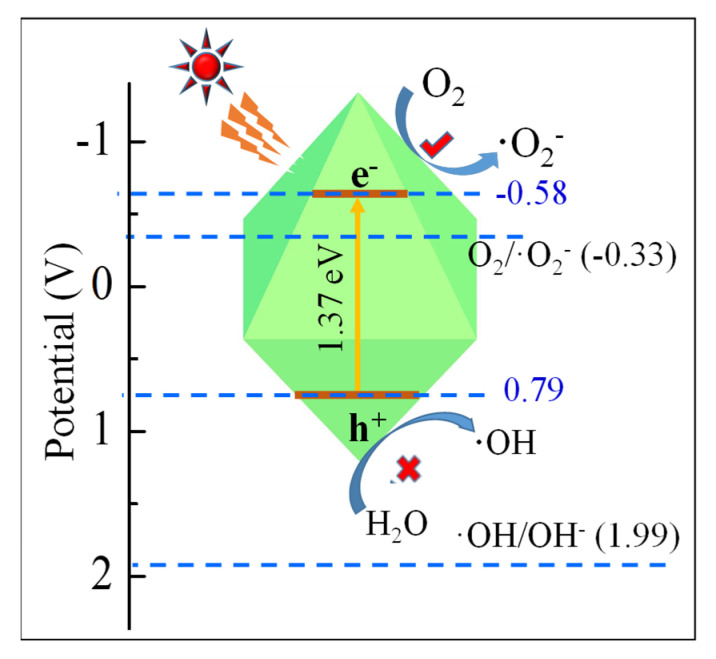
Photocatalytic mechanism of MnS for TCH removal.

The adsorption-photocatalysis mechanism of octahedral MnS was possible due to the sufficient vacant sites of unique octahedral structure and the narrow band gap structure (seen in Figure 8). Considering the zwitterionic and anionic species of TCH at pH > 3.3, TCH molecules were likely to adsorb on the active sites of octahedral MnS at a pH of 3.3~10.75 through the electrostatic attraction [12,61]. In addition, ECB value of MnS treated at 433 K for 16 h (−0.58 eV) was more negative than the redox potential value of O_2_/O^2−^ (−0.33 eV), while the corresponding ECB value (0.79 eV) was less positive than the redox potential value of OH/OH^−^ (1.99 eV). It is indicated that photo-induced e^−^ was favorable for the conversion of dissolved O_2_ into O^2−^, but the separated h+ was unsuitable for the reaction of OH^−^ into OH, which was confirmed by the stronger ESR intensity of O^2−^ in comparison to OH (Figure 7B) [5,64]. Therefore, MnS with a unique structure such as octahedron was a potential photocatalyst for the efficient degradation of antibiotics from wastewater.

**Figure 9:**
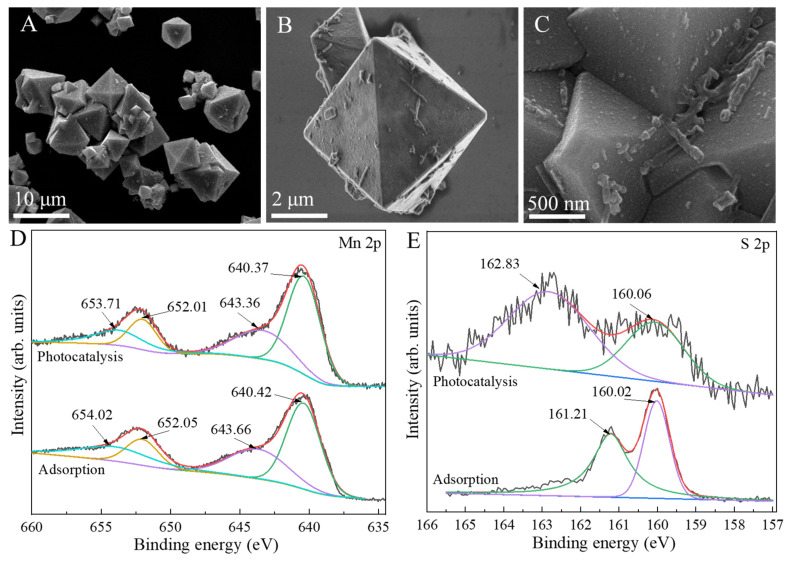
SEM images (**A**–**C**) and XPS spectra (**D**,**E**) of used MnS.

SEM images (Figure 9A–C) indicated that the morphology of used MnS after five cycles was not obviously affected in comparison to fresh samples (Figure 2D–F). However, the photo-corrosion seriously influenced the valence states of adsorbed and photo-catalyzed MnS. For comparison to fresh sample, the peaks of Mn 2p1/2 and Mn 2p3/2 of Mn^2+^ in adsorbed MnS shifted to 652.05 and 640.42 eV, and its corresponding satellite peaks changed to 654.02 and 643.66 eV, respectively (Figure 9D) [33,35]. The split peaks of Mn 2p in photo-catalyzed MnS shifted to the lower binding energies. In addition, the S 2p1/2 and S 2p3/2 peaks in adsorbed sample (Figure 9E) respectively changed to 161.21 and 160.02 eV, which were smaller than those of a fresh one. However, the S 2p1/2 and S 2p3/2 peaks in the photo-catalyzed sample located at 162.83 and 160.06 eV, respectively [37,38]. The change in valence states of Mn and S induced to the inferior adsorption-photocatalytic activity of MnS for TCH removal [42,43]. Hence, MnS-based heterojunctions were intensively fabricated for enhancing the utilization of solar light and photocatalytic durability.

## 3. Materials and Methods

### 3.1. Preparation of MnS

MnS was synthesized via a solvothermal route using ethylenediamine solution (C_2_H_8_N_2_, 20 vol%) as solvent. Typically, 3.0 mmol manganous nitrate (Mn(NO_3_)·4H_2_O), 4.0 mmol thiourea (CH_4_N_2_S), and 1.5 g polyvinylpyrrolidone (PVP, K30, *M*_w_ = 58,000) were dispersed into 40 mL above ethylenediamine solution under intensive stirring at room temperature for 40 min, and then poured into a 50 mL Teflon-lined autoclave for the heating treatment at 433 K for 16 h. After it was cooled down to room temperature, the above suspension was centrifuged, washed, and dried at 333 K to obtain MnS. MnS treated at 423, 443, and 453 K for 16 h as well as treated at 433 K for 14, 18, and 20 h were also prepared through the above process for comparison. The obtained samples were characterized by various technologies of Supplementary Materials.

### 3.2. Photocatalytic Experiment

The adsorption-photocatalytic performance of MnS was performed in a 200 mL Pyrex reactor with a 300 W Xe lamp with the detected power intensity of 500 mW cm^−2^. The irradiated distance and area of photocatalytic system were 20 cm and 20.42 cm^2^, respectively. A total of 20 mg MnS bulks were dispersed into 100 mL TCH solution of 260 mg L^−1^ and stirred at room temperature in the dark for 120 min to reach the balance of adsorption and desorption. After a certain time interval, 2 mL of the above solution was sampled, filtered, and detected on a high performance liquid chromatography (HPLC, Waters 2695). Effects of hydrothermal temperature, hydrothermal time, inorganic salts (such as FeCl_3_, CaCl_2_, NaCl, Na_2_SO_4_, and Na_3_PO_4_), and quenchers (such as p-BQ, KBrO_3_, EDTA-2Na, and t-BuOH) on the adsorption-photocatalytic activity of MnS were also performed.

## 4. Conclusions

The adsorption-photocatalytic of octahedral MnS for visible-light driven removal of TCH was intensively affected by the hydrothermal temperature, hydrothermal time, and inorganic salts. The optimal MnS treated at 433 K for 16 h could remove 94.83% of TCH solution of 260 mg L^−1^ within 180 min, and decreased to 89.68% after five cycles, exhibiting its superior adsorption-photocatalytic activity. This is attributed to the abundant vacant sites of octahedral structure for TCH removal and the suitable band-gap structure for radicals’ formation. The photo-induced e^−^ and O^2−^ played avital role in the MnS-assisted photocatalytic reaction.

## Data Availability

Not applicable.

## Supplementary Materials

The following supporting information can be downloaded at: https://www.mdpi.com/article/10.3390/ijms23169343/s1. References [47,65,66,67,68,69,70,71,72,73] are cited in the supplementary materials.
