# Supplementary material for: Efficient Adsorption-Photocatalytic Removal of Tetracycline Hydrochloride over Octahedral MnS
Source: Int J Mol Sci. 2022 Aug 19;23(16):9343. doi: 10.3390/ijms23169343 (PMC9408993; doi:10.3390/ijms23169343)
Supplement: Supplementary file 1 [file ijms-23-09343-s001.zip › ijms-1862514-supplementary.pdf]

Supported Information

Efficient Adsorption-Photocatalytic Removal of Tetracycline Hydrochloride over Octahedral MnS

Jing Guo [1], Tingting Liu [2], Hao Peng [1,*] and Xiaogang Zheng [2,*]

1 College of Chemistry and Chemical Engineering, Yangtze Normal University, Chongqing, 408100, China

2 College of Chemistry and Chemical Engineering, Neijiang Normal University，Neijiang Normal University, Neijiang, Sichuan 641100, China

* Correspondence: cqupenghao@126.com (H.P.); zhengxg123456@163.com (X.Z.)

S1. Materials

Manganous nitrate ($Mn(NO_3)\cdot4H_2O$), thiourea (CH_4N_2S), ethylenediamine ($C_2H_8N_2$), polyvinylpyrrolidone (PVP, K30, Mw=58,000), tetracycline hydrochloride (TCH, $C_{22}H_{25}ClN_2O_8$), p-benzoquinone (*p*-BQ, $C_6H_4O_2$), potassium bromate ($KBrO_3$), ethylenediamine tetraacetic acid disodium salt (EDTA-2Na, $C_{10}H_{14}N_2Na_2O_8\cdot2H_2O$), tert-butyl alcohol (*t*-BuOH, $C_4H_{10}O$), iron(III) chloride ($FeCl_3$), calcium chloride ($CaCl_2$), sodium chloride (NaCl), sodium sulfate (Na_2SO_4), andtrisodium phosphate anhydrous (Na_3PO_4)were analytical reagents, and purchased from Shanghai Aladdin Bio-Chem Technology Co., LTD (China) without further purification.

S2. Characterization

MnS wasevaluated via X-ray diffraction (XRD, Bruker D8) with a scanning speed of 5° min^{-1} and a scanning range of 20~ 80°using Cu Kα1 radiation operated at 40 kV and 40 mA. Field emission scanning electron microscopy (FE-SEM, Hitachi S-4800) was applied to detect the morphology and texture structure. The inductively coupled plasma optical emission spectrometer (ICP-OES, Optima 5300DV) was performed for investigating the actual content of metal compositions. X-ray photoelectron spectroscopy (XPS, Escalab 250XI) was conducted to investigate the surface chemical composition and bindingenvironment of samples referenced to the C 1s level at 284.8eV. UV–vis diffuse reflectancespectra (UV–vis DRS) were recorded at room temperature on aShimadzu UV-2600 spectrophotometer equipped with an integratingsphere using

$BaSO_4$ as the reflectance standard. The photoluminescence (PL) spectra weretested with a fluorescence spectrophotometer (FLSP 920, excitationat 380 nm).The signals of radical species generatedby the samples were detected on an electron spin resonance (ESR, Bruker A300, Germany) spectrometer at room temperature. The concentration of TCH was determined on a high performance liquid chromatography (HPLC, Waters 2695)with an C18 column (250×4.6 mm, 3 μm). In addition, their photoelectrochemical propertieswere evaluatedona photoelectric instrument (CEL-PECX2000, Beijing CEL Tech. Co., Ltd., China) equipped with a Vertex. C. EIS electrochemistry workstation (Ivium Technologies B.V., Holland) and a Xe lamp (λ> 420 nm, 240 mW cm^{-2}). In the three-electrode system of 0.1 mol L^{-1} Na_2SO_4 solution, the working electrode, counter electrode, and reference electrode are respectively Pt flake covered with 10 mg obtained photocatalyst film (0.5 cm^2), Ag/AgCl, and calomel electrode.

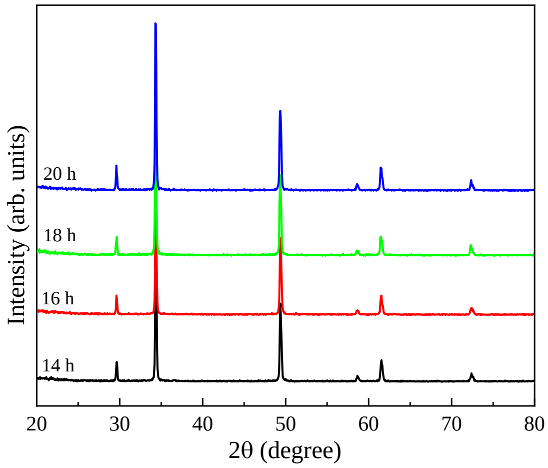

Figure S1. XRD patterns ofMnStreated with various time.

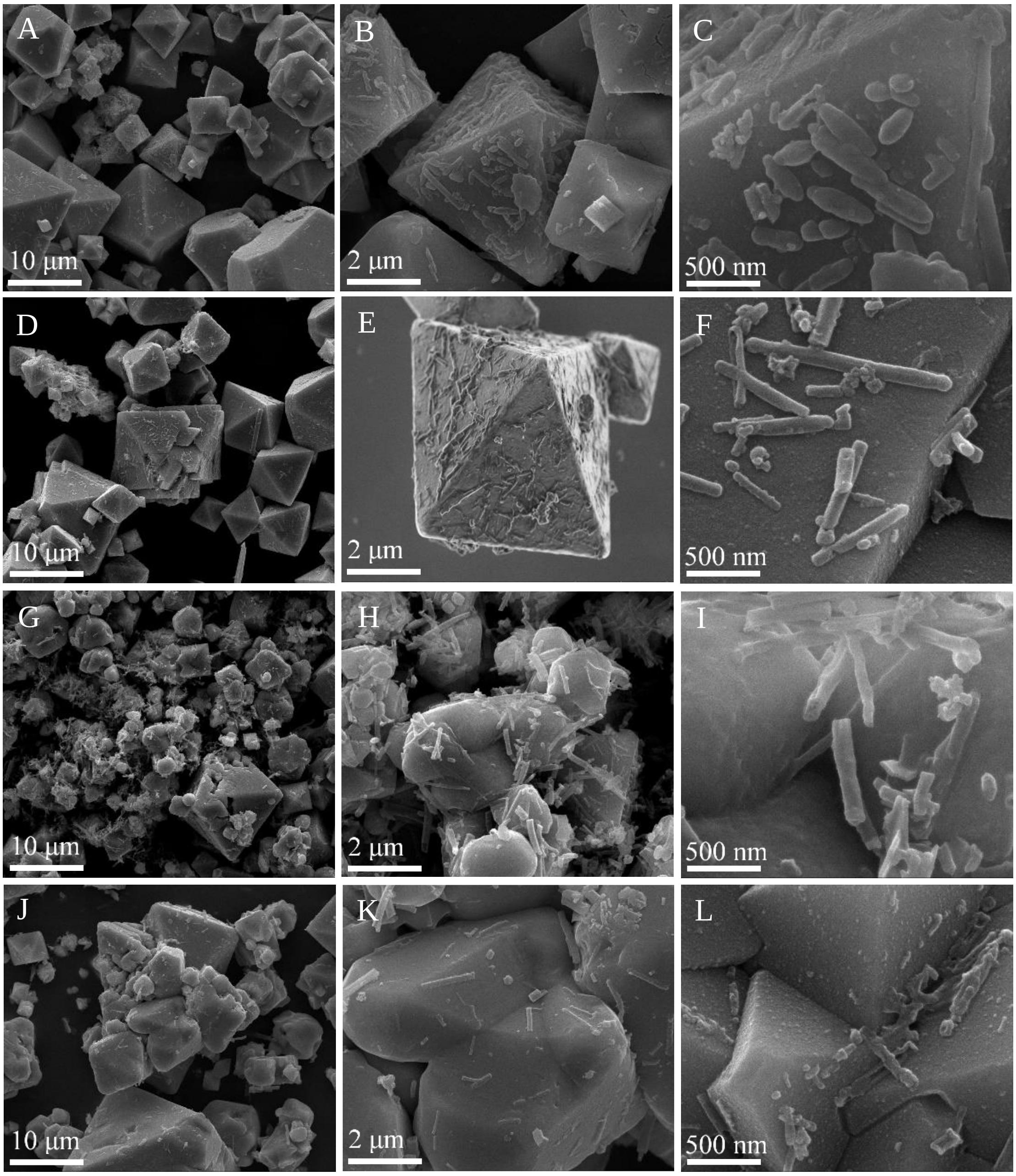

Figure S2. SEMimages ofMnStreated at 14 h (A-C),16 h (D-F), 18 h (G-I), and 20 h (J-L).

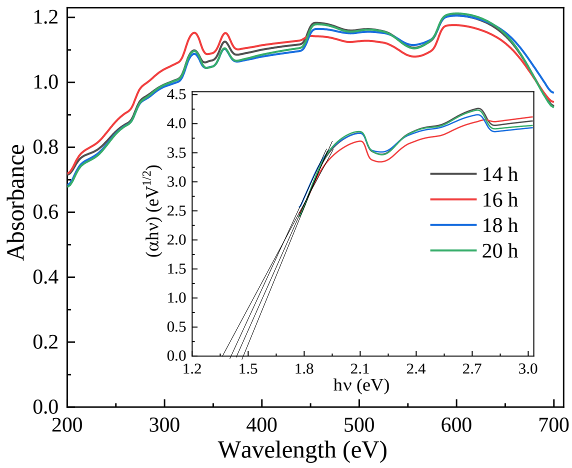

Figure S3. UV−vis DRS spectra of MnS treated with different hydrothermal time.

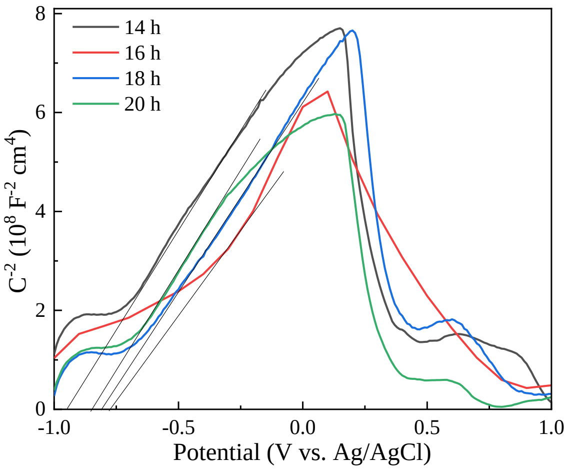

Figure S4. Mott-Schottky curves of MnS treated with different hydrothermal time.

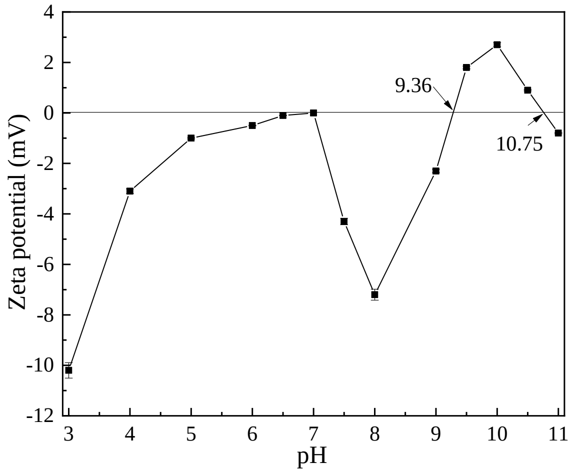

Figure S5. Zeta potential curveof MnS treated at 433 K.

Table S1. Surface parameters and electronic properties of MnScomposites.

T (K)	E_g (eV)	E_{FB} (V) [a]	E_{CB} (V) [a]	E_{VB} (V) [b]	t (h)	E_g (eV)	E_{FB} (V) [a]	E_{CB} (V) [a]	E_{VB} (V) [b]
423	1.45	−0.866	−0.67	0.78	14	1.44	−0.950	−0.75	0.69
433	1.37	−0.778	−0.58	0.79	16	1.37	−0.778	−0.58	0.79
443	1.52	−0.925	−0.73	0.79	18	1.41	−0.808	−0.61	0.80
453	1.57	−0.974	−0.78	0.79	20	1.49	−0.847	−0.65	0.84

[a] $E_{CB} = E_{FB} + 0.197$ (0.197 is the standard electrode potential of Ag/AgCl electrode).
[b] $E_{VB} = E_g + E_{CB}$.

Table S2. Comparison of adsorption-photocatalytic capacity of MnS and reported photocatalysts.

Photocatalysts	Lamp	Dosage ($g\ L^{-1}$)	Pollutant	Concentration ($mg\ L^{-1}$)	Time (min)	Removal efficiency (%)	Ref.
MnS/MoS_2	Xe (250 W)	0.2	Methylene blue	32	60	94	[1]
MnS/Ag-PVP	Xe (500 W)	0.015	Methylene blue	25	160	60	[2]
$Mn_{0.6}Zn_{0.4}Fe_2O_4@Zn_{1-x}Mn_xS$	Xe (300 W)	1	phenol	25	120	100	[3]
CdS@LDHs	Xe (500 W)	0.4	Tetracycline	50	300	93.04	[4]
$CuAl_2O_4/g\text{-}C_3N_4$	Xe (300 W)	0.2	Tetracycline	100	60	90	[5]
MnCuS	LED (24 W)	0.3	Orange II	70	120	98.2	[6]
$CuInS_2/Bi_2MoO_6$	Xe (300 W)	1.0	Tetracycline	50	120	84.7	[7]
WO_3/Bi_2MoO_6	Xe (500 W)	0.6	Tetracycline	20	180	85.9	[8]
$BaTiO_3/CF$	UV light	0.2	Tetracycline	20	180	96	[9]
$TiO_2@SCN$	Xe (300 W)	0.1	Tetracycline	10	60	98.1	[10]
MnS	Xe (300 W)	0.2	Tetracycline	260	180	94.83	This work

References

[1] X. Chen, J. Zhang, J. Zeng, Y. Shi, S. Lin, G. Huang, H. Wang, Z. Kong, J. Xi, Z. Ji, MnS coupled with ultrathin MoS2 nanolayers as heterojunction photocatalyst for high photocatalytic and photoelectrochemical activities, J. Alloy. Compd. 771 (2018) 364-372.

[2] A. Syed, N. Marraiki, S. Al-Rashed, A. Elgorban, M. Yassin, A potent multifunctional MnS/Ag-polyvinylpyrrolidone nanocomposite for enhanced detection of Hg^{2+} from aqueous samples and its photocatalytic and antibacterial applications, Spectrochim. Acta A 244 (2021) 118844.

[3] Z. Niu, X. Tao, H. Huang, X. Qin, C. Ren, Y. Wang, B. Shan, Y. Liu, Green synthesis of magnetically recyclable $Mn_{0.6}Zn_{0.4}Fe_2O_4@Zn_{1-x}Mn_xS$ composites from spent batteries for visible light photocatalytic degradation of phenol, Chemosphere 287 (2022) 132238.

[4] T. Dai, Z. Yuan, Y. Meng, B. Xie, Z. Ni, S. Xia, Performance and mechanism of photocatalytic degradation of tetracycline by Z–scheme heterojunction of CdS@LDHs, Appl. Clay Sci. 212 (2021) 106210.

[5] W. Chen, J. Huang, Z. He, X. Ji, Y. Zhang, H. Sun, K. Wang, Z. Su, Accelerated photocatalytic degradation of tetracycline hydrochloride over $CuAl_2O_4$/g-C_3N_4 p-n heterojunctions under visible light irradiation, Sep. Purif. Technol. 277 (2021) 119461.

[6] J. Zhou, H. Cheng, J. Ma, M. Peng, Y. Kong, S. Komarneni, Persulfate activation by MnCuS nanocomposites for degradation of organic pollutants, Sep. Purif. Technol. 261 (2021) 118290.

[7] J. Guo, L. Wang, X. Wei, Z. Alothman, M. Albaqami, V. Malgras, Y. Yamauchi, Y. Kang, M. Wang, W. Guan, X. Xu, Direct Z-scheme $CuInS_2/Bi_2MoO_6$ heterostructure for enhanced photocatalytic degradation of tetracycline under visible light, J. Hazard. Mater. 415 (2021) 125591.

[8] W. Han, T. Wu, Q. Wu, Fabrication of WO_3/Bi_2MoO_6 heterostructures with efficient and highly selective photocatalytic degradation of tetracycline hydrochloride, J. Colloid Interf. Sci. 602 (2021) 544–552.

[9] P. Demircivi, B. Gulen, E.B. Simsek, D. Berek, Enhanced photocatalytic degradation of tetracycline using hydrothermally synthesized carbon fiber decorated $BaTiO_3$, Mater. Chem. Phys. 241 (2020) 122236.

[10] K. Divakaran, A. Baishnisha, V. Balakuma, K. Perumal, C. Meenakshi, R. Kannan, Photocatalytic degradation of tetracycline under visible light using TiO_2@sulfur doped carbon nitride nanocomposite synthesized via in-situ method, J. Environ. Chem. Eng. 9 (2021) 105560.
